# Carotid body tumors: radioguided surgical approach

**DOI:** 10.1186/1756-9966-28-148

**Published:** 2009-12-10

**Authors:** Ombretta Martinelli, Luigi Irace, Rita Massa, Sara Savelli, Fabrizia Giannoni, Roberto Gattuso, Bruno Gossetti, Fabrizio Benedetti-Valentini, Luciano Izzo

**Affiliations:** 1 Department of Emergency, Institute of Vascular Surgery - "Umberto I" Hospital, University "Sapienza", Viale del Policlinico 155 - 00161, Roma, Italy; 2 Department of Nuclear Medicine, Institute of Radiology - "Umberto I" Hospital, University "Sapienza" - Viale del Policlinico 155 - 00161, Roma, Italy; 3 Department of Surgery, "P. Valdoni" Institute of Surgery - "Umberto I" Hospital, University "Sapienza" - Viale del Policlinico 155 - 00161, Roma, Italy; 4 Department of Vascular Surgery, Institute of Surgery "Umberto I" Hospital, University "Sapienza" Viale del Policlinico 155 - 00161, Roma, Italy

## Abstract

**Background:**

Carotid body tumours (CBTs) are very rare lesions which should be treated as soon as possible even when benign since small tumour size permits easier removal and lower incidence of perioperative complications and recurrence. Malignant forms are rare and they can be identified by lymph node invasion and metastases in distant locations. The need of reliable and effective diagnostic modalities for both primary CBTs and its metastases or recurrence is evident.

The present study reviews our experience and attempt to define the role of colour coded ultrasound (CCU) and Somatostatin receptor scintigraphy (SRS) with Indium-111-DTPA-pentetretide (Octreoscan^®^) using both planar and single photon emission tomography (SPECT) technique in the diagnosis and follow-up of these uncommon lesions within a multidisciplinary approach.

**Methods:**

From 1997 to 2008, 12 patients suffering from 16 CBTs (4 bilateral) were investigated by CCU and SRS-SPECT before and after surgery. All tumours were grouped according to Shamblin's classification in order to assess the technical difficulties and morbidity of surgical resection on the ground of their size and relationship with the carotid arteries. Intraoperative radiocaptation by Octreoscan^®^) was also carried out in all cases to evaluate the radicality of surgery. All perioperative scans were evaluated by the same nuclear medicine physician.

**Results:**

Preoperatively CCU showed CBTs (four were not palpable) with a sensitivity of 100%. Radioisotope imaging identified the CBTs as chemodectomas in 15 cases while no radioisotopic uptake was detected in 1 vagus nerve neurinoma. No evidence of metastasis or multicentricity were seen by total body radioisotopic scans. Combined data from CCU and SRS-SPECT allowed to determine tumour size in order to select 7 larger tumours which were submitted to selective preoperative embolization.

Intraoperatively Octreoscan demonstrated microscopic tumour leftovers promptly removed in 1 case and an unresectable remnant at the base of the skull in another case.

During follow-up CCI and radioisotope scans showed no recurrence in 14 cases and a slightly enlargement of the intracranial residual as detected during surgery in 1 patient.

**Conclusion:**

CCU may allow an early and noninvasive detection of CBTs and hence safer operations. The combined use of CCU and SRS-SPECT provide useful data to identify those tumours and to evaluate their extent and carotid arteries infiltration. Radioisotope imaging is a sensitive modality to detect metastases and lymph node involvement that are markers of CBT malignancy. After surgery CCU and SRS-SPECT can be accurate modalities for surveillance for an early detection of CBTs recurrence.

## Introduction

Carotid body tumours (CBTs) are rare neck tumours typically located at the carotid bifurcation. They are uncommon non chromaffin paragangliomas (PGs) and contain somatostatin receptor sites which enable localization by somatostatin receptor scintigraphy (SRS) with Indium-111-DTPA-pentetretide (Octreoscan^®^) using both planar and single photon emission tomography (SPECT) techniques; this modality allows to identify both the primary tumour, bilaterality, metastases in distant locations and recurrence which is reported in about 6% of cases [1] after surgery.

The main signs and symptoms of CBT include a slow growing pulsating mass at the level of carotid bifurcation and a peripheral cervical neuropathy related to the largest tumours but they may be clinically silent for a long time even when malignant.

The CBT is generally benign but also the benign forms have no true capsule and grow progressively, adhering to and encasing the vessels and nerves, compressing and dislocating the pharynx and even eroding the base of the skull; therefore they should never be left untreated even when they are supposed to be benign. In addition to the potential for adjacent tissue infiltration, they can be bilateral in up to 5% of cases [2]. Malignant form is suggested to be only around 5-10% [3] and only the lymph node invasion and metastases in distant locations (head, neck, chest, abdomen and pelvis) can identify the malignancy since mitotic figures or nuclear abnormalities may not always be found.

The natural history of those tumours can be unpredictable even for the benign ones and an early surgical excision at presentation is advisable since they may destroy glossopharingeal, vagal, hypoglossal and recurrent laryngeal nerves or invade the adjacent carotid arteries making the surgical management problematic according to Shamblin's clinicopathologic analysis [4].

Reliable and effective diagnostic methods for both primary CBTs and its metastases or recurrence are needed.

According to our previous experience and the data from literature [5,6], CBTs diagnosis can be carried out by colour coded ultrasound (CCU) at an early stage even before they become palpable. Computed tomography angiography with contrast medium administration (angio-CT) can further investigate both carotid arteries and CBTs and minimize the need for diagnostic conventional angiography that may be limited to those patients with indeterminate findings and within preoperative endovascular embolization of the afferent vessel performed to reduce tumor mass. Magnetic resonance angiography with contrast medium administration (angio-MR) is a reliable alternative to CT. Both angio-CT and angio-MR of the neck are sensitive to assess the presence of tumours at the carotid bifurcation and the relationship of the tumour with the adjacent structures but they do not provide data about the potential for malignancy and postoperative early recurrence because the tumors are too small with respect to their resolution power. As far as angio-CT concerns, it also causes a substantial exposure to ionizing radiations in a patient in which a total-body scanning has to be performed to detect potential metastases or multicentricity. MR angiography cannot be performed in patient with pacemaker or stainless stell prosthesis. Moreover those diagnostic modalities yield a risk of nephropaty and adverse effects due to contrast media administration. The nuclear medicine images obtained by SRS-SPECT have shown to be very accurate to determine the nature of the neck mass and to localize the CBTs; radioisotope scans also allow to detect areas of possible metastases throughout the body and to discover postoperative early recurrence.

The present study reviews our experience in perioperative use of CCU and SRS-SPECT for screening test, diagnostic confirmation and follow-up of CBTs within a multidisciplinary team approach in an effort to reduce the need of more invasive conventional imaging methods (CT, MR and angiography).

## Methods

From January 1997 to June 2008, 12 patients operated upon for 16 carotid body tumours (bilateral in 4 cases) were submitted to perioperative somatostatin receptor scintigraphies; before surgery and during follow-up the tracer used was indium-111-DTPA-pentetreotide (Ocreoscan ^®^) and the technique employed in this investigation was both planar and single photon emission tomography (SPECT); during surgery a gamma-probe was used.

The patient data taken into account were: age, gender, tumour size, bilaterality, postoperatively mortality and morbidity and recurrence during follow-up.

Average age was 51 years (range: 24-74 years) and 40% of patients were males.

CCU was performed as the first diagnostic approach in all patients with an Ultramark 9 ATL Philiphs equipment in the first part of this experience and with a Toshiba Aplio XP equipment successively. Typical ultrasound features included the presence of a solid hypoechoic vascular mass with a low-resistance flow pattern at Doppler frequency analysis, a hypervascular pattern at colour and power Doppler imaging; CCU also showed intrinsic carotid disease if present.

Neck angio-CT and angio-MR were combined to ultrasounds to define tumour feeding vessels, the relationship with the adjacent structures and the cranial extension in the neck for a better planning of the best surgical approach. Total body angio-CT was not performed to minimize the risks related to the high dose of radiation burden for CT.

Digital substraction carotid angiography (DSA) was carried out in those cases scheduled for endovascular preoperative embolization performed in order to reduce tumour vascularity and size; embolization was always followed by operation within 1 or 2 days. During DSA, contemporary balloon internal carotid blockade (Mata's test) was performed to determine the patient's tolerance to carotid cross-clamping. The sensitivity of this test was improved by the use of transcranial Doppler monitoring.

Preoperative total body SRS- SPECT was carried out by intravenous injection of 150 MBq 111In-pentetretide (StarCam 2000 at first and then StarCam 4000i). Nuclear scans included head, neck, chest, abdomen and pelvis and were repeated at 4 and 24 hours after injection with medium energy collimators and both 171 keV and 245 keV with a 15% window. The protocol included a 40-minute acquisition on 128 × 256 matrix. SPECT images were obtained by 30-minute acquisition on 64 × 64 matrix by using the same collimators.

All perioperative scans were evaluated by the same nuclear medicine physician.

If abnormal radioactivity was detected in other regions of the body than neck, nuclear scans would have been repeated for the same areas during the follow-up.

Table 1 summarizes the diagnostic methods employed for pre-operative evaluation in all cases.

**Table 1:** Preoperative investigation modalities in 16 CBTs

*Technique*	*n. CBTs (%)*
Color-coded imaging	16 (100%)

Indium 111In-pentreotide scintigraphy -SPECT*	16 (100%)

Angio-MR	7 (58.3%)

Angio-CT	9 (75%)

Digital selective angiography**	8 (66.6%)

* Body images were acquired from head, neck, chest, abdomen and pelvis

** Preoperative embolization was carried out in 7 out of 8 cases submitted to selective carotid angiography

When CCU and radioisotopic scans demonstrated a very high tumour site above the angle of the mandible, a multidisciplinary treatment involving vascular and maxillofacial teams was planned.

All tumours were grouped according to Shamblin's classification in order to assess the difficulty and morbidity of surgical resection: group I included all small tumours non yet adhering to the carotids; group II included larger tumours partially encasing the vessels and adhering the nerves whose dissection may cause nerve damage; group III included largest tumours completely encasing carotid arteries with a high danger for nerves and need for carotid resection and reconstruction.

Intraoperative radio-localization was carried out on all lesions by a hand-held gamma-detecting probe connected to a special counting unit (Octreoscan-Navigator-USSC) within 24 hours radiopharmaceutical administration by the same nuclear medicine physician than preoperative scanning. Radioactivity measurements were undertaken on the tumour in vivo compared with the background on the tumour bed to detect remnants and on lymph nodes to reveal invasion.

The carotid arteries were exposed through a standard cervicotomy, hypoglossal and vagus nerves were always identified and the common, internal and external carotid arteries were dissected. Resection was always attempted from the inferior margin of the tumour at the carotid bifurcation and extended onto the internal and external carotid arteries.

Preoperative CCU and radiosotopic scans suggested the need of a treatment involving vascular and maxillofacial teams in 4 patients and intraoperative findings confirmed the need of that multidisciplinary approach.

None of the 5 Shamblin's class I tumours required an internal carotid artery resection although in 1 case external carotid artery was interrupted; they all were fairly easily removed without neurological complications.

Ablation of the 5 CBTs in Shamblin's class II required: 2 external carotid artery resection, 1 carotid bifurcation PTFE patch angioplasty and 2 internal carotid artery replacement with a ASV graft.

At surgery all tumours of Shamblin's class III extended very high above the angle of the mandible and required digastric and pre-stilomastoid muscle resection plus vertical osteotomy of the mandibular ramus to get a wider space near the skull base. A forewarned maxillo-facial surgical team always resected and later reconstructed the mandibular bone in order to treat those CBTs.

A CBTs ablation with carotid arteries resection and internal carotid artery replacement (2 PTFE-TW and 2 ASV grafts) was carried out in all cases combined to external carotid artery resection in 2.

The patient suffering from vagus nerve neurinoma had the nerve resection; in another case vagus, hypoglossal and superior laryngeal nerves interruption was mandatory to allow complete removal of adhering tumours.

The pathologic examination of the tumour and sampling of jugular lymph nodes were carried out in all cases.

Follow-up included CCU examination and radioisotopic scans at 1, 6 and 12 months postoperatively and every 12 months after the first year to show local recurrence and to control arterial reconstruction. When any abnormal tracers of CBTs were identified, CT or MR scans from those areas were obtained to confirm.

## Results

The CCU failed in a sharp evaluation of tumour size and its superior level in the neck in 2 cases (13.3%) when compared with CT and MR techniques data and with Octreoscan SPECT imaging.

**Figure 1:**
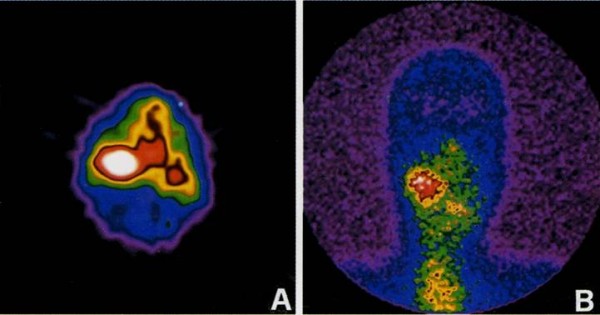
**A) Markedly increased focal tracer uptake in the right cervical region in both planar and B) SPECT scans due to a massive chemodectoma at the right carotid bifurcation**.

Preoperatively, In-111 pentectreotide uptake by nuclear scans (Figure 1) was high in all tumours detected by ultrasounds but one that was a neurinoma originating from vagus nerve as confirmed intraoperatively and by histological data.

Compared with SRS-SPECT, CCU showed a good diagnostic accuracy with a sensitivity and a specificity of 100% and 93.7% respectively.

**Figure 2:**
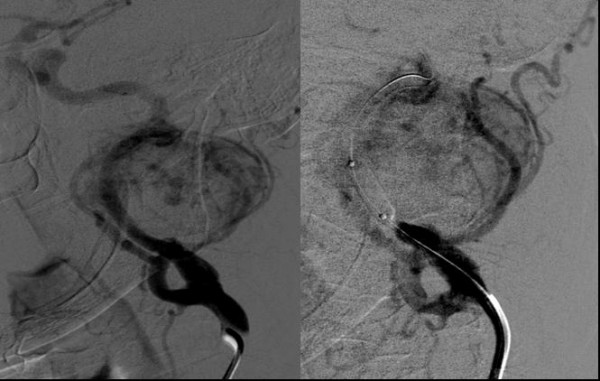
**Conventional angiography showing a carotid body tumor (left) and its selective embolization (right)**.

Preoperatively ultrasounds data and radioisotopic scan findings were combined to group CBTs on the ground of their estimated size and their relationship with the adjacent vessels (Table 2). On the ground of preoperative size measurement, CBTs embolization was carried out for the largest 3 tumors of group II and for the 4 CBTs of group III (43.7%) and led to shrinkage of tumour and reduction of its vascularity in 6 out of 7 cases (85.7%) (figure 2).

**Table 2:** Preoperative classification of CBTs on ground of size measurements and relationship with adjacent vessels on CCU and radioisotopic scans (111In-pentetreotide scintigraphy -SPECT)

Group	Numper of patients	Mean size on CCU	Mean sixe on radioisotopic sacns	of CBTs on the ground of size measurements and relationship with adjacent vessels on CCU	of CBTs on the ground of size measurements and relationship with adjacent vessels on radioisotopic scans
I	5	16 mm	18 mm	well defined	not adhering

II	5	28 mm	31 mm	partially defined	partially adhering

III	5	43 mm	47 mm	undefined	strongly adehering

At surgery 5 CBTs were classified on size as Shamblin's class 1 and they all could be easily dissected from carotid arteries since they didn't adhere to the carotid arteries, 5 were in Shamblin's class 2 and partially encircled carotid bifurcation; the remaining 5 tumours were in class 3 since they were strongly adherent to carotid vessels and surgical resection in a periadventitial plane was not possible. Table 3 summarizes intraoperative measurements of all tumours; they ranged from 1.4 to 2.7 cm for CBTs in class I (mean size 2.0 cm), from 1.8 to 3.6 cm for class II (mean size 2.7 cm) and from 4.5 to 5.1 cm for class III (mean size 5 cm).

**Table 3:** Intraoperative Shamblin's classification and size of CBTs

*Shamblin's class*	*n°*	*Size range*	*Mean size*
I	5	1.4-2.7 cm	2.0 cm

II	5	1.8-3.6 cm	2.9 cm

III	5	4.5-5.1 cm	5.0 cm

Preoperative CCU tumor measurements agreed with intraoperative Shamblin's classification. Preoperative CCU and radioisotopic scans suggested the need of a treatment involving vascular and maxillofacial teams for 4 patients and that multidisciplinary approach was confirmed to be useful by intraoperative findings.

**Figure 3:**
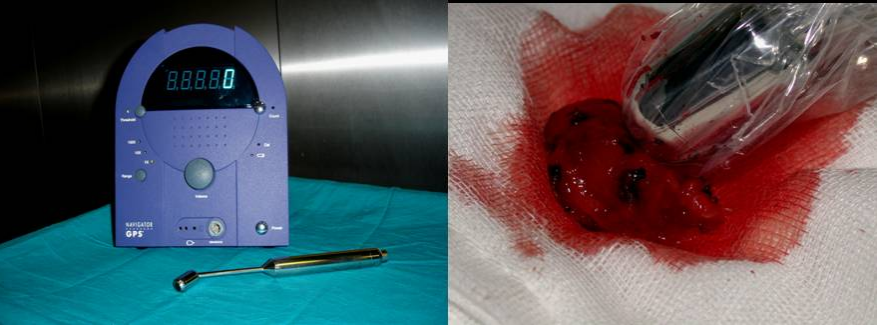
**A) The gamma probe and meter system used in all patients in our study**. B) its intraoperative use.

During surgery gamma probe (figure 3) showed no radiotracer uptake from the neurinoma and identified all CBTs which had more than twofold radioisotopic uptake as compared to background (mean tumor/background ratio: 3.02).

After removal, by means of radioactivity measurement in the tumour bed a small leftovers of tumour tissue partially encasing the internal carotid artery wall was discovered and required a more accurate resection followed by carotid bifurcation PTFE angioplasty in 1 case (6.6%).

In another case radiotracer uptake by an unreseactable remnant was recorded at the base of the skull not even detected by other subsequent imaging methods (6.6%) performed during follow-up.

Radioactivity measurements on lymph nodes never revealed tumour invasion.

The pathologic results confirmed the diagnosis of CBT in 15 cases and showed no metastasis both in jugular lymph nodes and carotid arteries. Lymph nodes sampling showed no residual disease.

**Figure 4:**
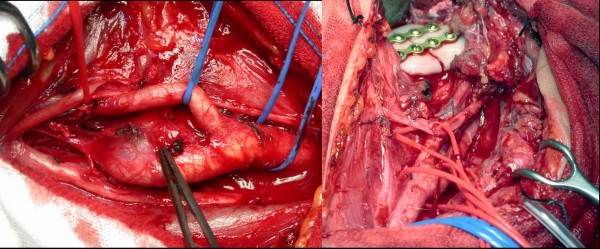
**A) Intraoperative image showing a carotid body tumor at carotid bifurcation**. B) The same case after resection and reconstruction of the mandibular bone.

Perioperative mortality was nil. No intraoperative brain ischemia occurred. Deviation of tongue was seen after surgery in 3 cases (21%) but disappeared in a few days. Five patients (30%) sustained permanent cranial nerve injuries causing dysphonia in 3 case that was associated with dysphagia in 1 and with dysphagia and total tongue deviation in another case. Postoperative course was uneventful in all cases. (figure 4)

During follow-up (from 4 months to 10 years; median 3.6 years) clinical, CCU and Octreoscan SPECT of carotid arteries were performed at 6 and 12 months after surgery and yearly thereafter.

These controls showed no signs of recurrence in all cases. Nuclear scan confirmed the presence of the intracranial remnant in 1 case as detected intraoperatively which slightly enlarged without clinical evidence within the following 8 years making further CT or MR controls unnecessary.

## Discussion

Since the first report in 1891 [7], there have been a large number of sporadic reports in literature concerning carotid body tumours. CBT is bilateral in approximately 5% of cases and 33% of the sporadic and familial forms respectively [8] and it usually presents as a gradually enlarging mass that is incidentally identified.

Although malignant forms of those tumours are suggested to be only around 5%, the early surgical excision of CBTs at presentation is mandatory because of their locally invasive nature and the uncertainty about their natural history [9]. They may grow unpredictably and destroy glossopharingeal, vagal, hypoglossal and or recurrent laryngeal nerve [10]. They can also invade the adjacent carotid arteries making surgical management problematic and indicating the need of CBTs as soon as the diagnosis is established.

The larger the tumour the more difficult is the resection, and the more neural and vascular injuries occur, so the diagnosis of CBTs should be as earlier as possible. Lack of clinical diagnosis has been reported in up to 30% of patients since these neoplasm can be confused with enlarged lymph nodes or brachial cysts or salivary glands. The advent of new imaging modalities allow their detection at an earlier stage even before they become clinically evident. CT or MR angiography (MR) are reliable diagnostic techniques to evaluate CBTs and their potential multicentricity or recurrence. The main concerns about CT are the need of contrast medium administration related to potential adverse effects (eg. acute renal failure) and radiation burden with their inherent risks. MR angiography cannot be performed when patient has pace maker or stainless stell prosthesis. Further limitation to the use of that modality is the risk of nephropaty and nephrogenic systemic fibrosis due contrast medium administration. These drawbacks make those imaging techniques unfit for preclinical screening and long-term follow-up of CBTs.

In our experience CCU proved to be useful and very sensitive for detection of CBTs before the onset of symptoms; it also allows the differential diagnosis with other neck mass avoiding ill-advised biopsy. Our experience is consistent with those of several series [11,12] that indicate Duplex scanning as a non-invasive method for screening evaluation of even small tumours and for their subsequent earlier treatment. This is a crucial point since available reports suggest cranial nerves and vessels injures are more likely related to locally advanced disease rather than operative techniques.

Ultrasounds study alone may fail in a precise evaluation of size and superior level in the neck of larger tumours when compared with angio-CT and intraoperative measurements [13]. In our series CCU could establish a definitive diagnosis to proceed with surgery only for tumours less than 2 cm while required further adjunctive instrumental techniques for larger neoplasms. Both CCD and radiological imaging didn't provide any information for differential diagnosis between chemodectomas and vagus nerve neurinoma that was obtained by 111In-pentetreotide scintigraphy -SPECT scans. Moreover combination of CCU evaluation and 111In-pentreotide scintigraphy -SPECT scans may help not only to localize the suspected paragangliomas at neck but also to determine their nature, size and involvement of adjacent structures on the ground of the tumour's somatostatin receptors. Earlier hystologic studies indicated an high incidence of CBT malignancy on the ground of their mitotic activity, capsular invasion and pleomorphism but the reports by Martin [14] and Westerband [15] suggest that CBT's metastasis to regional lymph nodes, brain, bone, liver and lungs are the actual hallmark of malignancy; they occur from 5 to 10% and can be reliably detected by radioisotope scans.

In our series radioisotopic scan allowed to exclude potential multicentricity and metastasis of CBTs in an accurate fashion [16,17] and it is far less invasive than total body angio-CT scanning as far as radiation exposure and contrast media toxicity concern [18].

In our study a good correlation between preoperative classification based on CCU imaging and radioisotopic measurement and Shamblin's intraoperative classification was found.

Data from CCU and radioisotopic investigations allowed to plan a multidisciplinary treatment for Shamblin II and III CBTs which encase and or infiltrate carotid arteries and other adjacent structures making dissection difficult even in the benign forms.

CCU and nuclear evaluation also provided useful information for selective preoperative embolization. According with other authors [19], we believe that the apparent benefits of embolization should be weighed against the risk of stroke and that procedure should be limited to infiltrating tumours greater than 3 cm in diameter; an accurate pre-operative evaluation by ultrasounds and nuclear methods can be useful for selection of greater and more invasive tumours to be treated by embolization. A further advantage of the early detection and resection of smaller lesion is the lower need of preoperative embolization and its attendant risks [20].

Additionally a reliable radioisotopic evaluation of the distal extension of tumours above the angle of the mandible suggest the need of a combined surgical team of maxillofacial and vascular surgery for the distal internal carotid exposure as high as possible at the skull base by mandibulotomy within a multidisciplinary team treatment of this disease to reduce the incidence rate of peripheral neurological complications that can occur during the resection of all CBTs.

The risk of tumour recurrence is related to minimal leftovers which can be missed by surgical resection [21]. Intraoperative gamma probe radioactivity measurement on the tumour in vivo compared with the background on the tumour bed allows to detect tiny remnants so that even the smallest ones can be readily identified and removed. These remnants may be removed by a more radical radioguided revision of carotid arteries and resection of adjacent tissues. Radiotracer uptake shows also inoperable residuals that need a careful surveillance during follow-up [22].

During follow-up serial controls by ultrasounds and Octreoscan SPECT may be used to evaluate carotid arteries reconstruction and to detect the recurrence of tumour at the level of carotid bifurcation in the effort to reduce the need of more invasive CT or MR controls. Nuclear controls has also showed to be a reliable modality to follow the growing of unresectable residuals not detectable by CCU.

## Conclusion

Even for benign forms, resection of whole CBTs is recommended as soon as the diagnosis is established because of their unpredictable local invasive behaviour. Advances in diagnostic modalities based on ultrasounds and radioisotope imaging have increased earlier discovery of those tumours even before they become palpable. The nuclear images obtained by Octreoscan SPECT is shown to be very accurate to determine the nature of the neck mass and to localize the CBTs; SPECT scan also allows to detect areas of potential postoperative early recurrence.

A reliable preoperative evaluation of tumour details concerning their size, extent and relationship with adjacent vessels can be obtained by combining the two techniques and allow to plan when a multidisciplinary approach should be used to treat these patients involving the fields of vascular surgery, otolaryngology, maxillofacial and radiology. The early detection and an accurate measurements of larger lesions also provide an additional advantage by decreasing the need for preoperative embolization and its attendant risks. An early diagnosis permits an earlier treatment of smaller CBTs minimizing the risk of cranial nerves and vessels injures.

Radioactivity measurements performed during surgery is helpful to detect leftovers of tumour tissue, even the smallest ones which could be missed without the help of Octreoscan. Since even tiny remnants may lead to recurrence, intraoperative radionucleotide investigation can better define the outcome of surgery.

During follow-up, CCU and radioisotope imaging combined together are sensitive and less invasive methods to detect potential recurrence and to monitor growth progression of unresectable remnants of "these curious little tumors" as defined by F.B. Lund [23].

## Competing interests

The authors declare that they have no competing interests.

## Authors' contributions

OM and LI carried out the color coded ultrasonographic studies, participated in the sequence alignment and drafted the manuscript. RM carried out the Somatostatin receptor scintigraphy (SRS) with Indium-111-DTPA-pentreotide. SS, LI participated in the sequence alignment. MFG, RG and BG participated in the design of the study and performed the statistical analysis. FBV conceived of the study, and participated in its design and coordination. All authors read and approved the final manuscript.

## References

[B1] Nora JD Hallett JW O'Brien PC Naessens JM Cherry KJ Jr Pairolero PC Surgical resection of carotid body tumors: long-term survival, recurrence and metastasis Mayo Clin Proc 1988 63 348 52 3352318 10.1016/s0025-6196(12)64856-3

[B2] Farr HW Carotid body tumors: a 40 year study Cancer 1980 30 260 5 10.3322/canjclin.30.5.260 6250680

[B3] Hammond SL Greco DL Lambert AT McBiles M Patton GM Indium-In 111 penetreotide scintigraphy: application to carotid body tumors J Vasc Surg 1997 25 905 8 10.1016/S0741-5214(97)70221-0 9152319

[B4] Shamblin WR ReMine WH Sheps SG Harrison EG Carotid body tumor (chemodectoma): clinicopathologic analysis of 90 cases Am J Surg 1971 122 732 9 10.1016/0002-9610(71)90436-3 5127724

[B5] Sajid MS Hamilton G Baker DM Joint Vascular Research Group A multicenter review of carotid body tumour management Eur J Vasc Endovasc Surg 2007 34 2 127 30 10.1016/j.ejvs.2007.01.015 17400487

[B6] Luna-Ortiz K Rascon Ortiz M Villavicencio-Valencia V Granados Garcia M Herrera-gomez Carotid Body tumors review of 20 year experience Oral Oncology 2005 41 56 61 10.1016/j.oraloncology.2004.06.006 15598586

[B7] Dias Da Silva A O'Donnel S Gillespie D Goff J Shriver C Rich N Malignant Carotid body tumor: a case report J Vasc Surg 2000 32 4 821 3 10.1067/mva.2000.107766 11013048

[B8] Saldana MJ Salem LE Travezan R High altitude hypoxia and chemodectomas Hum Pathol 1973 4 251 63 10.1016/S0046-8177(73)80012-7 4706179

[B9] Fruhwirth J Kock G Hauser S Gutschi S Beham A Kainz J Paragagliomas of the carotid bifurcation: oncological aspects of vascular surgery Eur J Surg Oncol 1996 22 88 92 10.1016/S0748-7983(96)91748-5 8846876

[B10] Lack EE Carotid body paraganglioma Washington DC Armed Force Institute of Pathology 1997 231 42

[B11] Muhm M Polterauer P Gstottner W Temmel A Richling B Undt G Diagnostic and therapeutic approaches to carotid body tumors Arch Surg 1997 132 279 84 9125028 10.1001/archsurg.1997.01430270065013

[B12] Koopmans KP Jager PL Kema IP Kerstens MN Albers F Dullaart RP 111In-octreotide is superior to 123I-metaiodobenzylguanidine for scintigraphic detection of head and neck paragagliomas J Nucl Med 2008 49 8 1232 7 10.2967/jnumed.107.047738 18632829

[B13] Kasper GC Welling RE Wladis AR Cajocob DE Grisham AD Tomsick TA Gluckman JL Muck PE A multidisciplinary approach to carotid paragagliomas Vasc Endovasc Surg 2006 40 6 467 74 10.1177/1538574406290254 17202093

[B14] Martin CE Rosenfeld L Mc Swain B Carotid body tumors: a 16-years follow-up of seven malignant cases South Med J 1973 66 1236 43 4745602 10.1097/00007611-197311000-00009

[B15] Westerband A Hunter GC Cintora I Coulthard SW Hinni ML Gentile AT Devine J Mills JL Current trends in the detection and management of carotid body tumors J Vasc Surg 1998 28 1 84 92 10.1016/S0741-5214(98)70203-4 9685134

[B16] Smith JJ Passman MA Dattilo JB Guzman RJ Naslund TC Nerreville JL Carotid body tumor resection: does the need for vascular reconstruction worsen outcome? Ann Vasc Surg 2006 20 4 435 9 10.1007/s10016-006-9093-0 16786441

[B17] Ozay B Kurc E Orhan G Yucel O Senay S Tasdemir M Gorur A Aka SA Surgery of carotid body tumor: 14 cases in 7 years Acta Chir Belg 2008 108 1 107 11 18411584

[B18] Litle VR Reilly LM Ramos TK Preoperative embolization of carotid tumors: when is appropriate? Ann Vasc Surg 1996 10 5 464 8 10.1007/BF02000594 8905066

[B19] Robinson JG Shagets FW Becket WC Spies JB A multidisciplinary approach to reducing morbidity and operative blood loss during resection of carotid body tumor Surgery Gynecology and Obstetrics 1989 168 166 70 2911794

[B20] Baskoyannis KC Georgopoulos SE Klonaris CN Tsekouras NS Felekouras ES Pikoulis EA Griniatsos JE Papalambros El Bastounis EA Surgical treatment of carotid body tumors without embolization Int Angiol 2006 25 40 5 16520723

[B21] Kollert M Minovi AA Draf W Bockmühl U Cervical Paragangliomas--Tumor Control and Long-Term Functional Results after Surgery Skull Base 2006 16 4 185 191 17471317 10.1055/s-2006-950386 PMC1766458

[B22] Filippi L Benedetti Valentini F Gossetti B De Vincentis G Scopinaro F Massa R Intraoperative gamma probe detection of head and neck paragangliomas with 111In-pentreotide: a pilot study Tumors 2005 91 2 173 6 10.1177/030089160509100213 15948547

[B23] Lund FB Tumors of the carotid body JAMA 1917 69 348 352

